# Supplementary material for: METTL3-mediated chromatin contacts promote stress granule phase separation through metabolic reprogramming during senescence
Source: Nat Commun. 2024 Jun 26;15:5410. doi: 10.1038/s41467-024-49745-5 (PMC11208586; doi:10.1038/s41467-024-49745-5)
Supplement: Supplementary file 1 — Supplementary Information [file 41467_2024_49745_MOESM1_ESM.pdf]

METTL3-mediated chromatin contacts promote stress granule phase separation through metabolic reprogramming during senescence

Chen et al., Supplementary Information

Supplementary Table 1 | The number of intra-chromosome loops based on H3K27Ac HiChIP at indicated conditions.

Category	Control	Sen/shControl	Sen/shMETTL3	Sen/shMETTL14
**Valid Intra-chromosomal interactions < 5Kb**	1600348	1632385	2018608	2520297
**Valid Intra-chromosomal interactions 5Kb-2Mb**	10740250	8548989	12107137	17792516
**Valid Intra-chromosomal interactions > 2Mb**	3800054	3495911	4502529	7009019

Supplementary Table 2 | Overlapped 38 genes for Venn Diagram as in Fig. 1d.

Number	RefseqID	Gene Symbol
**1**	NM_000189	*HK2*
**2**	NM_000576	*IL1B*
**3**	NM_000584	*CXCL8*
**4**	NM_000641	*IL11*
**5**	NM_000758	*CSF2*
**6**	NM_000921	*PDE3A*
**7**	NM_000963	*PTGS2*
**8**	NM_001034841	*ITPRIPL2*
**9**	NM_001098672	*HEPHL1*
**10**	NM_001145368	*PTPN3*
**11**	NM_001150	*ANPEP*
**12**	NM_001205019	*GK*
**13**	NM_001271213	*SQOR*
**14**	NM_001277742	*CYP26B1*
**15**	NM_001282485	*PRKG2*
**16**	NM_001718	*BMP6*
**17**	NM_001898	*CST1*
**18**	NM_002006	*FGF2*
**19**	NM_002090	*CXCL3*
**20**	NM_002422	*MMP3*
**21**	NM_002425	*MMP10*
**22**	NM_002426	*MMP12*
**23**	NM_002993	*CXCL6*
**24**	NM_002994	*CXCL5*
**25**	NM_004079	*CTSS*
**26**	NM_004267	*CHST2*
**27**	NM_007036	*ESM1*
**28**	NM_012413	*QPCT*
**29**	NM_020820	*PREX1*
**30**	NM_024524	*ATP13A3*
**31**	NM_130830	*LRRC15*
**32**	NM_139072	*DNER*
**33**	NM_178031	*TMEM132A*
**34**	NM_178429	*LCE2C*
**35**	NM_198041	*NUDT6*
**36**	NR_046035	*CXCL1*
**37**	NR_132768	*SNORA86*
**38**	NR_152420	*FBXL21*

Supplementary Table 3 | The number of intra-chromosome loops based on METTL3 HiChIP at indicated conditions.

Category	Control	Senescence
**Valid Intra-chromosomal interactions < 5Kb**	1710479	1668949
**Valid Intra-chromosomal interactions 5Kb-2Mb**	10818425	8894510
**Valid Intra-chromosomal interactions > 2Mb**	4105769	4514028

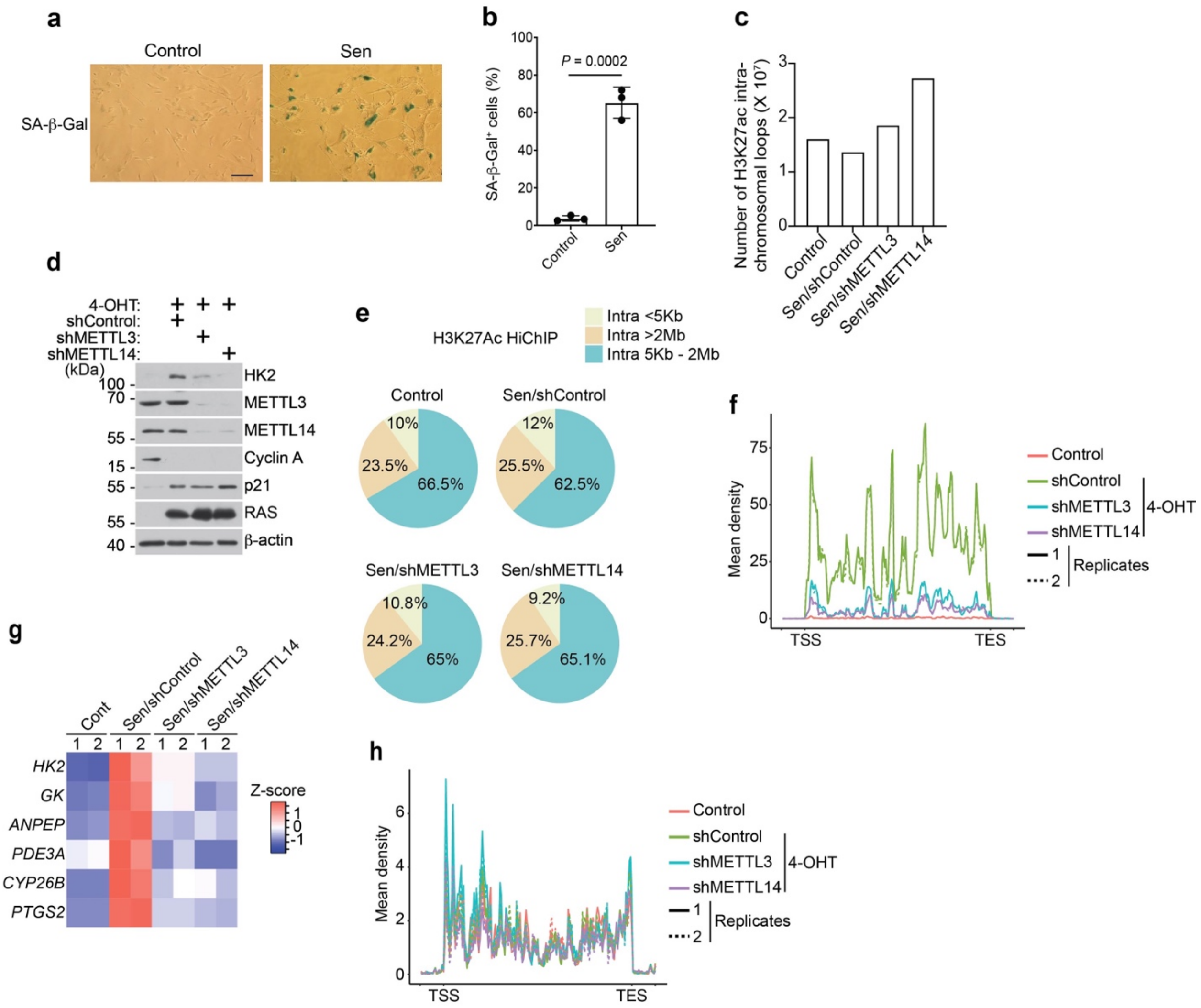

Supplementary Fig. 1 | SASP and metabolic genes are transcriptionally regulated by MTC. a, SA-β-Gal (beta Galactosidase) staining in Control and Senescent (Sen) cells, indicating sufficient induction of cellular senescence by 4-OHT. Scale bar, 50 µm. **b**, Quantification of SA-β-Gal positive cells as shown in **a**. **c**, Numbers of intra-chromosomal paired-end tags (PETs) from H3K27Ac HiChIP were compared across indicated conditions. **d**, Western blot showing the expression of the indicated proteins under indicated conditions. **e**, Pie diagram revealed the distribution of intra-chromosome interactions from H3K27Ac HiChIP at indicated conditions. **f**, Mean density profiles of fastGRO reads for SASP genes under indicated conditions. **g**, Heatmap revealed expression profiles for metabolic genes such as *HK2*, *GK*, ANPEP, *PDE3A*, *CYP26B*, and *PTGS2* at indicated conditions. Red represents higher expression, while blue represents lower expression, **h**, Mean density profiles of fastGRO reads for top expressed 40 genes under indicated conditions. Source data are provided as a Source Data file.

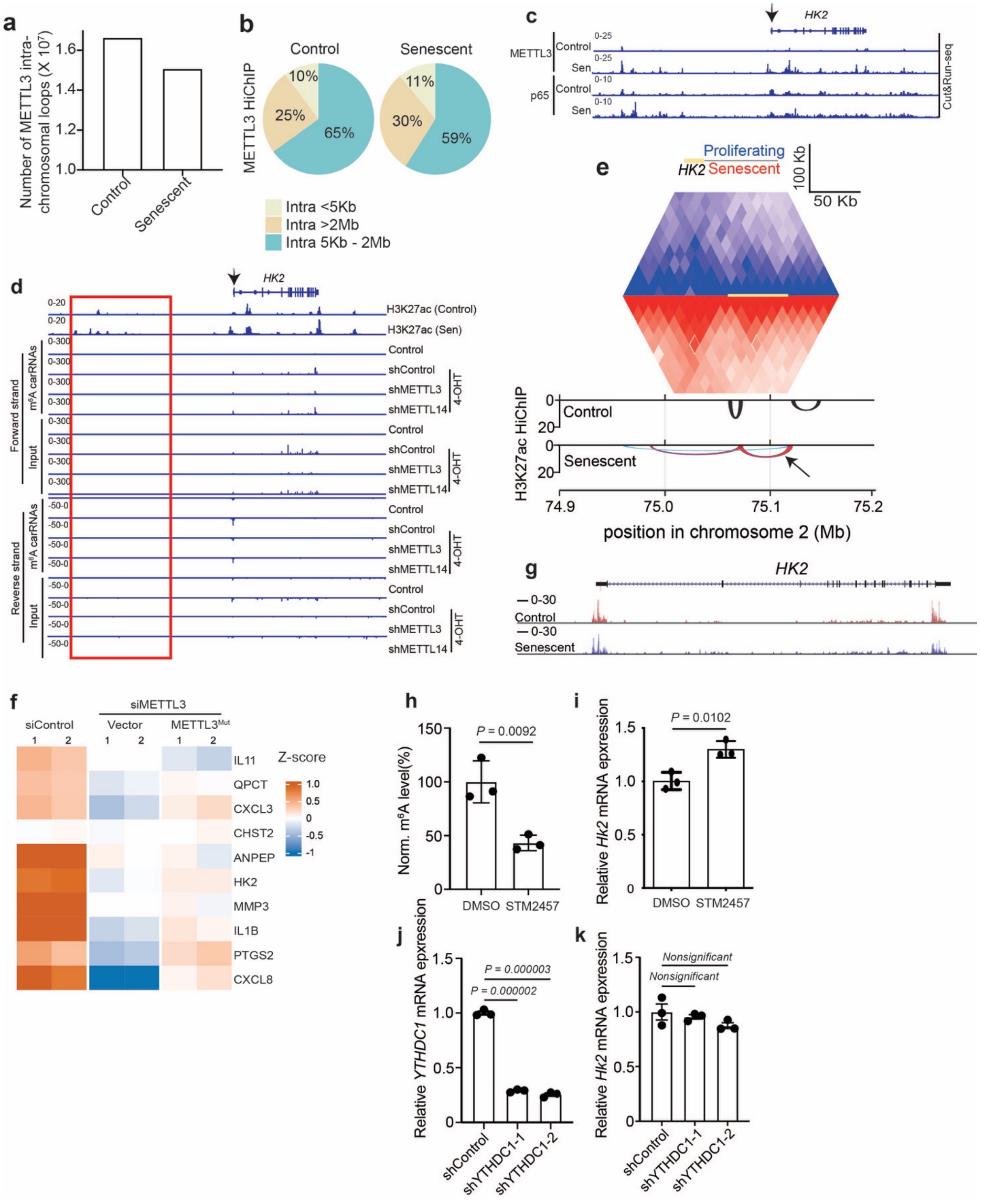
a
Number of METTL3 intra-chromosomal loops (X 10^7)
Control
Senescent
b
METTL3 HiChIP
Control
Senescent
10%
25%
65%
11%
30%
59%
Intra <5Kb
Intra >2Mb
Intra 5Kb - 2Mb
c
HK2
METTL3
p65
Control
Sen
Cut&Run-seq
d
HK2
H3K27ac (Control)
H3K27ac (Sen)
Forward strand
Reverse strand
m6A carRNAs
Input
Control
shControl
shMETTL3
shMETTL14
4-OHT
e
Proliferating
HK2 Senescent
100 Kb
50 Kb
H3K27ac HiChIP
Control
Senescent
position in chromosome 2 (Mb)
g
HK2
Control
Senescent
f
siMETTL3
siControl
Vector
METTL3Mut
Z-score
IL11
QPCT
CXCL3
CHST2
ANPEP
HK2
MMP3
IL1B
PTGS2
CXCL8
h
Norm. m6A level(%)
P = 0.0092
DMSO
STM2457
i
Relative Hk2 mRNA epxression
P = 0.0102
j
Relative YTHDC1 mRNA epxression
P = 0.000003
P = 0.000002
shControl
shYTHDC1-1
shYTHDC1-2
k
Relative Hk2 mRNA epxression
Nonsignificant
Nonsignificant

Supplementary Fig. 2 | HK2 is upregulated by MTC in senescence. **a**, Numbers of intra-chromosomal paired-end tags (PETs) from METTL3 HiChIP were compared across indicated conditions. **b**, Pie diagram revealed the distribution of intra-chromosome interactions from METTL3 HiChIP at indicated conditions. **c**, Tracks of publicly available METTL3 Cut&Run-seq and p65 Cut&Run-seq (GSE141992 [https://www.ncbi.nlm.nih.gov/geo/query/acc.cgi?acc=GSE141992]) showing peaks along *HK2* genomic region in RAS-induced senescent cells and proliferating control cells. Arrow indicates the transcription start site (TSS) of *HK2*. **d**. m^{6}A tracks along *HK2* genomic loci for both forward and reverse strands. Boxes indicated H3K27ac modification sites in control and senescent cells used to identify the regulatory chromatin region. **e**, Hi-C interaction frequency heatmap in senescent cells (below) compared to proliferating cells (upper) at a 10 kb resolution. Analysis was performed based on publicly available dataset (GSE118494 [https://www.ncbi.nlm.nih.gov/geo/query/acc.cgi?acc=GSE118494]). Rectangles point to the potential chromatin contacts of *HK2*. Hi-C interactions was aligned to the loop formation from H3k27Ac HiChIP. **f,** Heatmap showing relative expression of 10 MTC-regulated genes depicted in Fig. 1g under indicated conditions**.** Red represents higher expression, while blue represents lower expression. **g,** Tracks of publicly available m^{6}A-seq (GSE141993 [https://www.ncbi.nlm.nih.gov/geo/query/acc.cgi?acc=GSE141993]) shows the distribution of peak signals within *HK2* genomic locus in control and senescent cells. The m^{6}A signal was normalized to the corresponding input and the relative fold change is shown. **h**, Measurement of m^{6}A levels in senescent cells treated with DMSO or 2.5 μM METTL3 inhibitor (STM2457) for 48 hrs. **i**, Quantitative reverse transcription polymerase chain reaction (RT-qPCR) analysis of *HK2* expression level in senescent cells treated with DMSO or 2.5 μM METTL3 inhibitor (STM2457). **j**-**k**, RT-qPCR analysis of *YTHDC1* (**j**) and *HK2 (**k**)* expression level in senescent cells upon YTHDC1 knockdown. Data represent the mean ± SD of n=3 biological independent experiments. *P* values were calculated using a two-tailed Student's *t*-test (**e**). Source data are provided as a Source Data file.

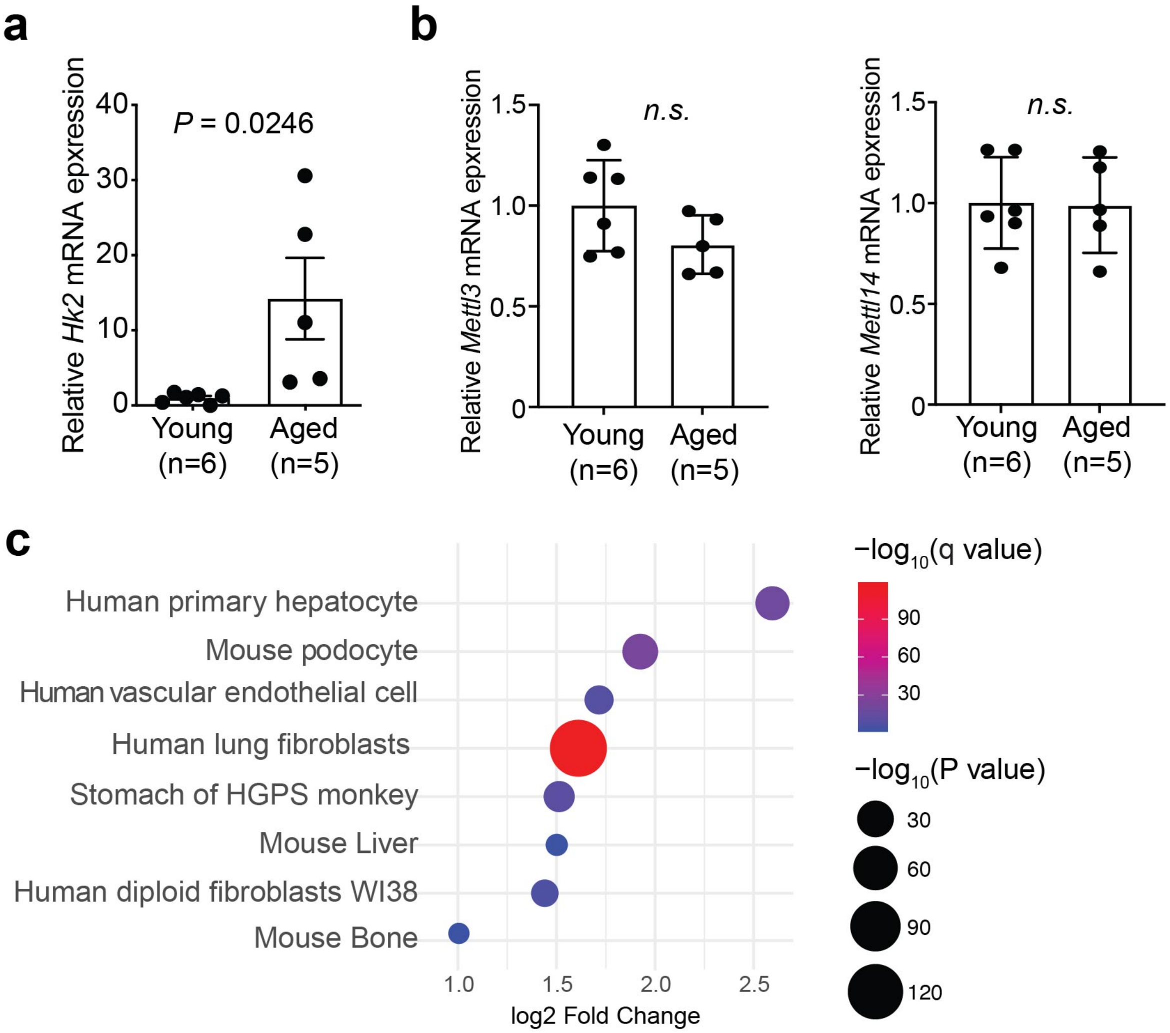

Supplementary Fig. 3 | Hk2 is upregulated in old hepatocytes than the young. a, Normalized Fragments Per Kilobase of transcript per Million mapped reads (FPKM) values of *Hk2,* from RNA-seq data of young (n=6) and aged (n=5) hepatocytes. **b**, Normalized Fragments Per Kilobase of transcript per Million mapped reads (FPKM) values of *Mettl3 and Mettl14,* from RNA-seq data of young (n=6) and aged (n=5) hepatocytes. **c**, Analysis of expression level of *HK2* (or *Hk2*) among different kinds of cells (senescent compared to proliferating) and tissues (aged compared to young) was performed through RNA-seq examination via Aging Atlas. *P* values were calculated using a two-tailed Student's *t*-test (**a**, **b**). Source data are provided as a Source Data file.

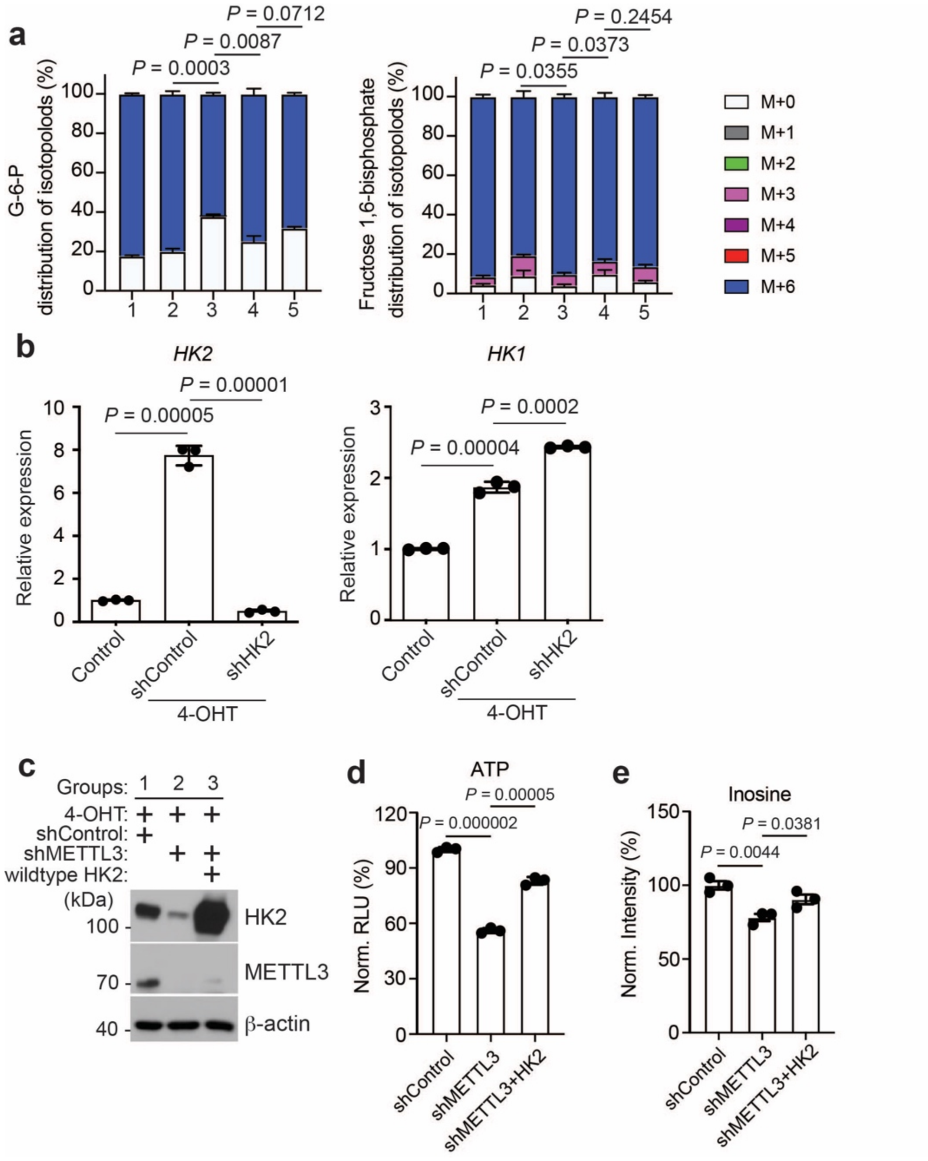

Supplementary Fig. 4 | ^{13}C Glucose isotope tracing reveals activated purine metabolism that can be regulated by HK2 in senescence. **a**, ^{13}C Glucose isotope tracing reveals fractional distribution of isotopologues for Glucose-6-phosphate (G-6-P) and Fructose 1,6-bisphosphate. 1, control proliferating cells. 2, RAS-induced senescent cells via 4-OHT with control shRNA (shControl). 3, senescent cells with shRNA targeting HK2 (shHK2). 4, senescent cells with shRNA targeting HK2 (shHK2) and rescued with wildtype Flag-HK2. 5, senescent cells with shRNA targeting HK2 (shHK2) and rescued with mutant Flag-HK2. **b**, RT-qPCR analysis of *HK2* and *HK1* expression level was performed in proliferating and senescent with or without HK2 KD. Data represent mean ± SEM (**a**) or SD (**b**) of n=3 biologically independent experiments. **c**, Western blot showing the expression of the indicated proteins under indicated conditions. **d**-**e**, ATP and inosine intracellular levels were measured under indicated conditions. Data represent mean ± SD of n=3 biologically independent experiments. *P* values were calculated using a two-tailed Student’s *t*-test (**a**, **b**). Source data are provided as a Source Data file.

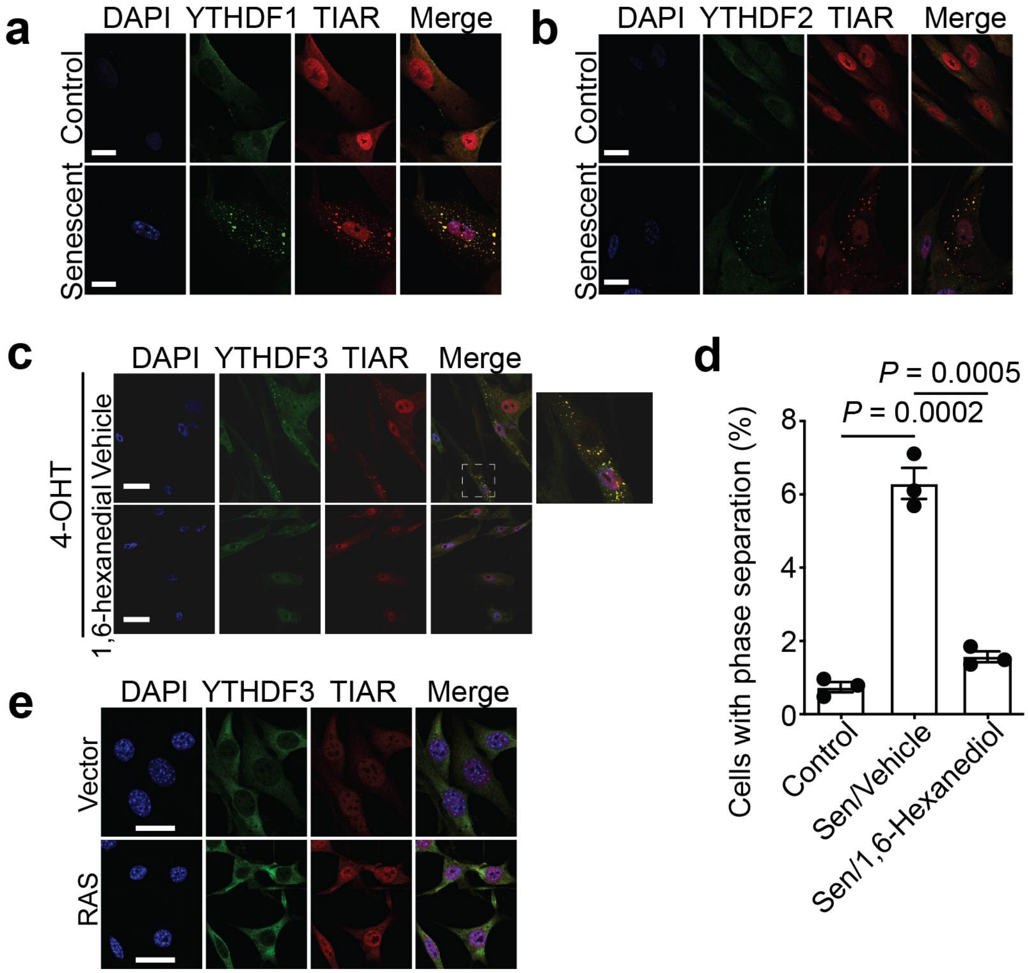

Supplementary Fig. 5 | Phase separation occurs in senescence. a-b, IF of control and RAS-induced senescent cells with antibodies targeting YTHDF1 (**a**) or YTHDF2 (**b**) and TIAR. **c**, IF of senescent cells with antibodies targeting YTHDF3 and TIAR with or without 7.5% 1,6-hexanediol treatment for 5 min. Rectangle represented the area that was shown on the right with 4X magnification, showing cases of phase separation. **d**, Quantification of the percentage of cells with phase separation events at indicated conditions was determined (n > 200 cells over three independent experiments). *P* values were calculated using a two-tailed Student's *t*-test. Error bars represent mean with SD. **e**, IF of control and RAS-induced NIH-3T3 transformed cells with antibodies targeting for YTHDF3 and TIAR. DAPI counterstaining was used to visualize the nuclei (**a**, **b**, **c**, **e**). Scale bars, 20 μm (**a**, **b**, **c**) and 25 μm (**e**). Source data are provided as a Source Data file.

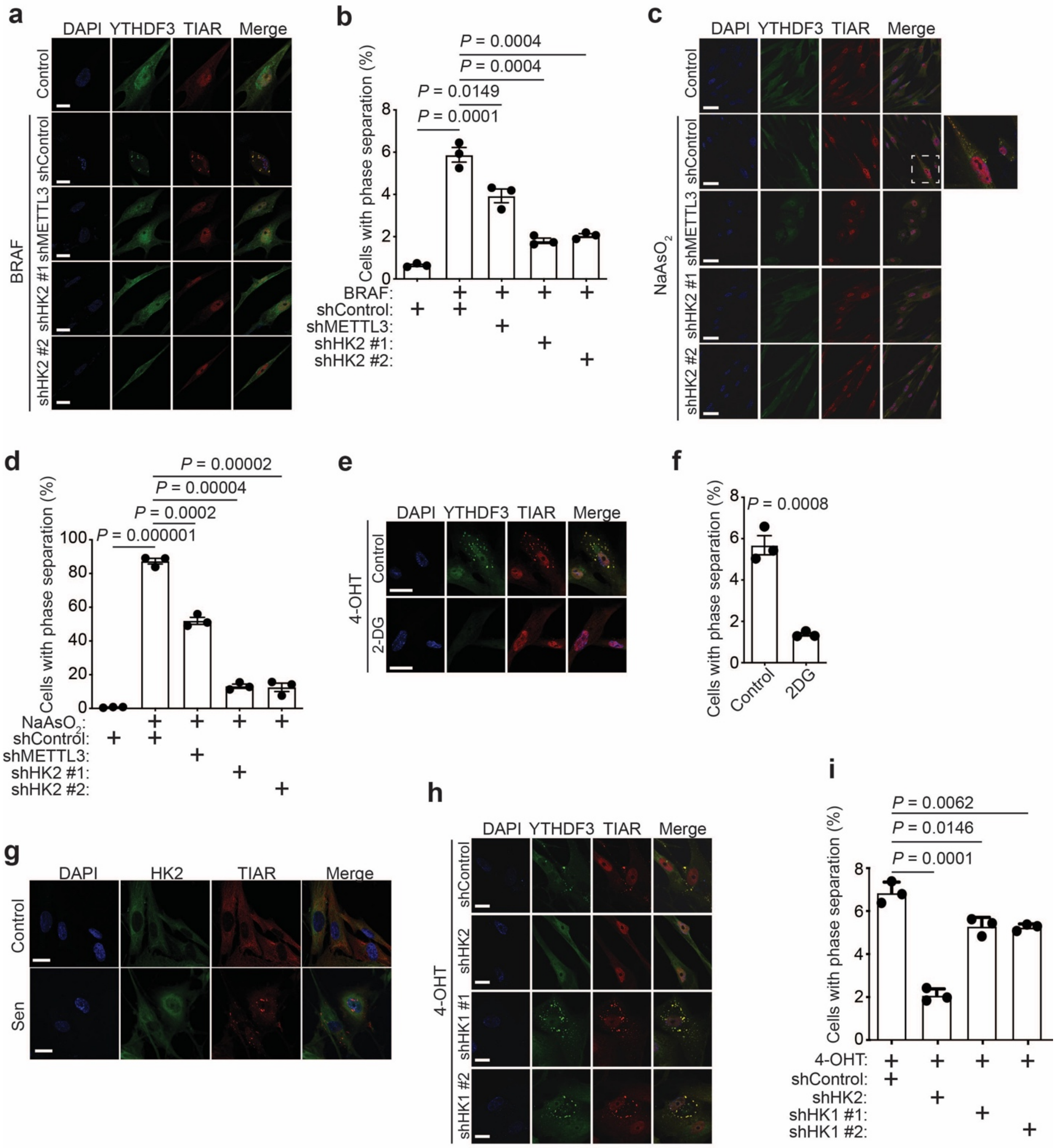
a
DAPI
YTHDF3
TIAR
Merge
Control
BRAF
shControl
shMETTL3
shHK2 #1
shHK2 #2
b
P = 0.0004
P = 0.0004
P = 0.0149
P = 0.0001
Cells with phase separation (%)
BRAF:
shControl:
shMETTL3:
shHK2 #1:
shHK2 #2:
c
NaAsO2
d
P = 0.00002
P = 0.00004
P = 0.0002
P = 0.000001
NaAsO2:
e
4-OHT
2-DG
f
P = 0.0008
Control
2DG
g
HK2
Sen
h
shHK2
shHK1 #1
shHK1 #2
i
P = 0.0062
P = 0.0146
P = 0.0001
4-OHT:
shHK2:
shHK1 #1:
shHK1 #2:

Supplementary Fig. 6 | HK2 promotes phase separation in senescence. **a**, IF of control and BRAF-induced senescent cells with antibodies targeting YTHDF3 and TIAR at indicated conditions. **b**, Quantification of the percentage of cells with phase separation events at indicated conditions was determined (n > 200 cells over three independent experiments). *P* values were calculated using a two-tailed Student's *t*-test. Error bars represent mean with SD. **c**, IF of IMR90 cells with antibodies targeting YTHDF3 and TIAR with or without exposure of 0.5 mM $NaAsO_2$ for 30 min to induce oxidative stress at indicated conditions. Rectangle represented the area that was shown on the right with 4X magnification, showing cases of phase separation. **d**, Quantification of the percentage of cells with phase separation events at indicated conditions, as in **c**, was determined (n > 200 cells over three independent experiments). *P* values were calculated using a two-tailed Student's *t*-test. Error bars represent mean with SD. **e**, IF of RAS-induced senescent cells with antibodies targeting YTHDF3 and TIAR when treated with solvent control and 10 mM 2-DG for 24 hrs. **f**, Quantification of the percentage of cells with phase separation events at indicated conditions, as in **e**, was determined (n > 200 cells over three independent experiments). *P* values were calculated using a two-tailed Student's *t*-test. Error bars represent mean with SD. **g**, IF of proliferating and RAS-induced senescent cells with antibodies targeting HK2 and TIAR. **h**, IF of RAS-induced senescent cells with antibodies targeting YTHDF3 and TIAR with control shRNA (shControl), shRNA targeting HK2 (shHK2) and shRNA targeting HK1 (shHK1 #1 and shHK1 #2). **i**, Quantification of the percentage of cells with phase separation events at indicated conditions, as in **h**, was determined (n > 200 cells over three independent experiments). *P* values were calculated using a two-tailed Student's *t*-test. Error bars represent mean with SD. DAPI counterstaining was used to visualize the nuclei (**a**, **c**, **e**, **g**, **h**). Scale bars, 20 μm (**a**, **c**, **e**, **g**, **h**). Data represent mean ± SD (**b**, **d**, **f**, **i**). *P* value was calculated using a two-tailed Student's *t*-test (**b**, **d**, **f**, **i**). Source data are provided as a Source Data file.

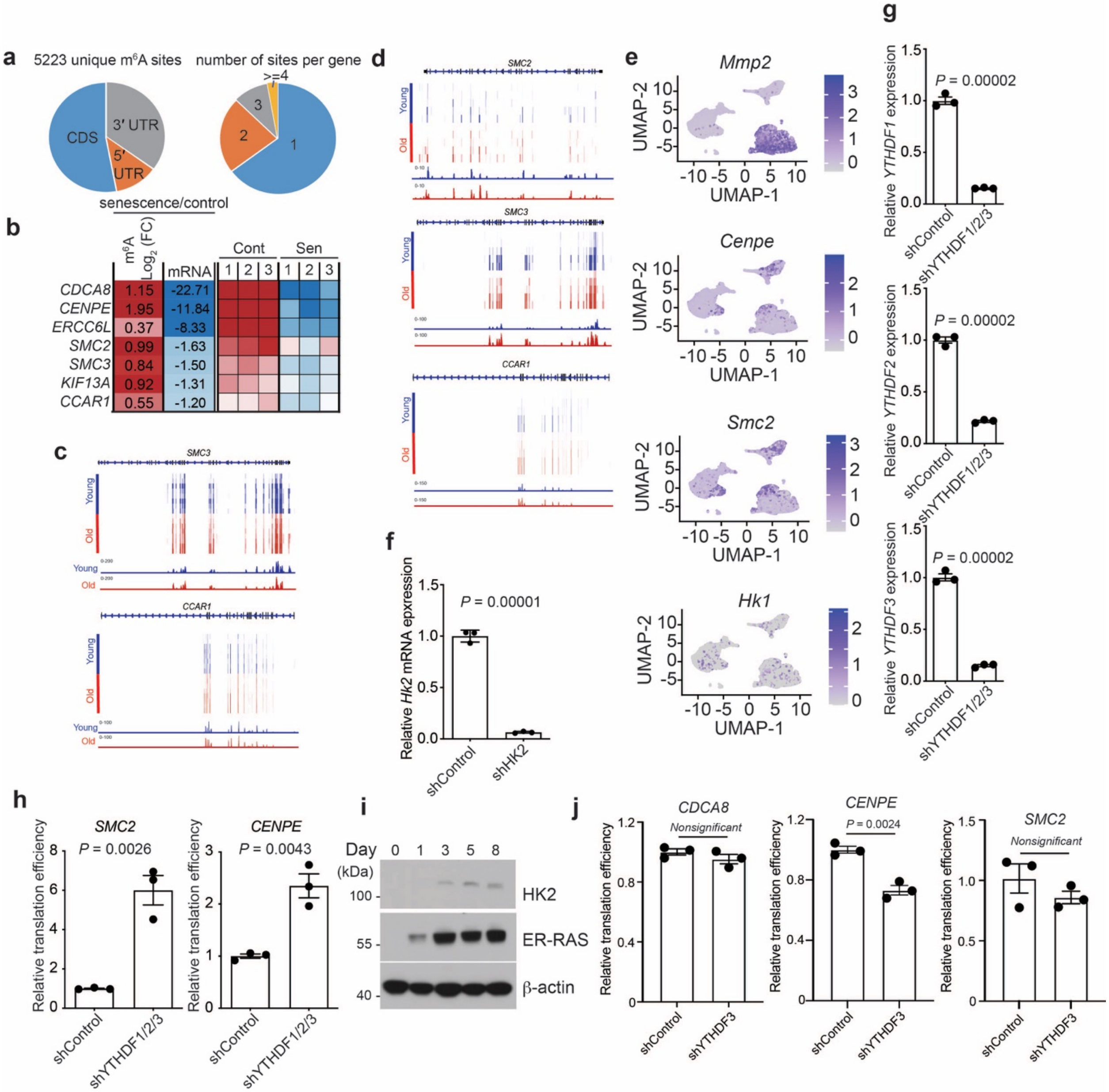
a
5223 unique m6A sites
CDS
3′ UTR
5′ UTR
number of sites per gene
1
2
3
>=4
b
senescence/control
m6A Log2 (FC)
mRNA
Cont
Sen
CDCA8 1.15 -22.71
CENPE 1.95 -11.84
ERCC6L 0.37 -8.33
SMC2 0.99 -1.63
SMC3 0.84 -1.50
KIF13A 0.92 -1.31
CCAR1 0.55 -1.20
c
SMC3
CCAR1
Young
Old
d
SMC2
SMC3
CCAR1
Young
Old
e
Mmp2
Cenpe
Smc2
Hk1
UMAP-1
UMAP-2
f
Relative Hk2 mRNA epxression
P = 0.00001
shControl
shHK2
g
Relative YTHDF1 expression
Relative YTHDF2 expression
Relative YTHDF3 expression
P = 0.00002
shControl
shYTHDF1/2/3
h
SMC2
P = 0.0026
CENPE
P = 0.0043
Relative translation efficiency
shControl
shYTHDF1/2/3
i
Day 0 1 3 5 8
(kDa)
HK2
ER-RAS
β-actin
j
CDCA8
Nonsignificant
CENPE
P = 0.0024
SMC2
Nonsignificant
Relative translation efficiency
shControl
shYTHDF3

Supplementary Fig. 7 | HK2 maintains stable growth arrest in senescence through sequestering cell-cycle related mRNAs containing polymethylated m^{6}A signals. a, The distribution of 5223 unique m^{6}A sites among protein coding sequence (CDS), 5' untranslated region (5' UTR), and 3' untranslated region (3' UTR) in senescence were identified from the publicly available m6A-seq (GSE141993 [https://www.ncbi.nlm.nih.gov/geo/query/acc.cgi?acc=GSE141993]). Numbers of m^{6}A peak signals was calculated for each gene. **b**, Heatmap reveals decreased gene expression of cell-cycle related genes including *CDCA8*, *CENPE*, *ERCC6L*, *SMC2*, *SMC3*, *KIF13A* and *CCAR1* in correspondence with its increased m^{6}A signals in senescence versus control cells. Log_2(FC), Log_2 (Fold Change). Red represents higher expression, while blue represents lower expression. **c-d**, Heatmap from publicly available m^{6}A-seq [CRA005942 (https://ngdc.cncb.ac.cn/gsa/browse/CRA005942)] revealed the distribution of the m^{6}A peak signals of *SMC2*, *SMC3*, and *CCAR1* from young (n=8) and aged (n=8) liver tissues (**c**) or young (n=5) and aged (n=5) heart tissues (**d**) in primates. Representative tracks show the median m^{6}A peak signals from either young or aged group. The m^{6}A signal was normalized to the corresponding input. **e**, UMAP projection were shown by Feature Plots depicting *Mmp2*, *Cenpe*, *Smc2*, and *Hk1* expression in single cells. **f**, RT-qPCR analysis of the expression of *HK2* in senescent cells with control shRNA (shControl) and shRNAs targeting HK2 (shHK2). **g**, RT-qPCR analysis of the expression of YTHDF1, YTHDF2, and YTHDF3 in senescent cells with control shRNA (shControl) and shRNAs targeting YTHDF1, YTHDF2, and YTHDF3 (shYTHDF1/2/3). **h**, Translation efficiency of SMC2 and CENPE was detected by polysome profiling in senescent cells with control shRNA (shControl) and shRNA targeting YTHDF1, YTHDF2, and YTHDF3 (shYTHDF1/2/3). Translational efficiency is normalized to free mRNA level for each gene. **i**, Western blot of time course experiment showing the expression of HK2 in 4-OHT induced senescence. **j**, Translation efficiency of *CDCA8*, *CENPE*, and *SMC2* was detected by polysome profiling with control shRNA (shControl) and shRNA targeting YTHDF3 before HK2 activation. Translational efficiency is normalized to free mRNA level for each gene. Data represent mean ± SD of n=3 biologically independent experiments (**f**, **g**, **h**, **j**). *P* value was calculated using a two-tailed Student's *t*-test (**f**, **g**, **h**, **j**). Source data are provided as a Source Data file.

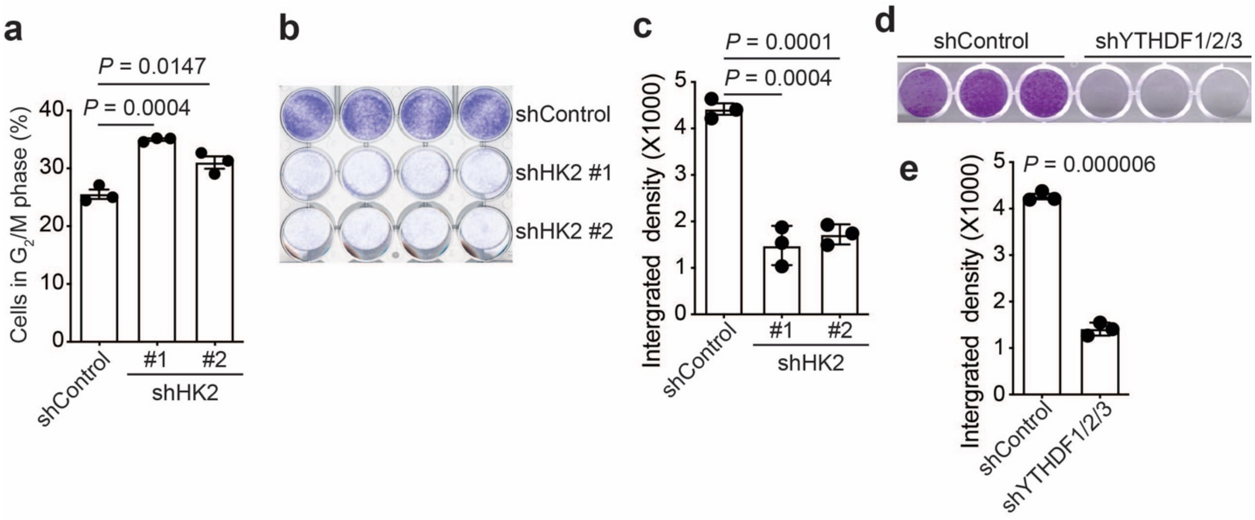

Supplementary Fig. 8 | HK2 maintains stable cell growth arrest of senescent cells. **a**, Cell cycle analysis by flow cytometry was performed in senescent cells with control shRNA (shControl) and shRNA targeting HK2 (shHK2 #1 and shHK2 #2). **b-c**, Cell viability was monitored through staining with crystal violet (**b**) in senescent cells with control shRNA (shControl) and shRNA targeting HK2 (shHK2 #1 and shHK2 #2) and the intensities of staining, as in **b,** was quantified (**c**). **d-e**, Cell viability was monitored through staining with crystal violet in senescent cells with control shRNA (shControl) and shRNA targeting YTHDF1, YTHDF2, and YTHDF3 (shYTHDF1/2/3) and the intensities of staining, as in **d,** was quantified (**e**). Data represent mean ± SD of n=3 biologically independent experiments (**a**, **c**, **e**). *P* value was calculated using a two-tailed Student's *t*-test (**a**, **c**, **e**). Source data are provided as a Source Data file.

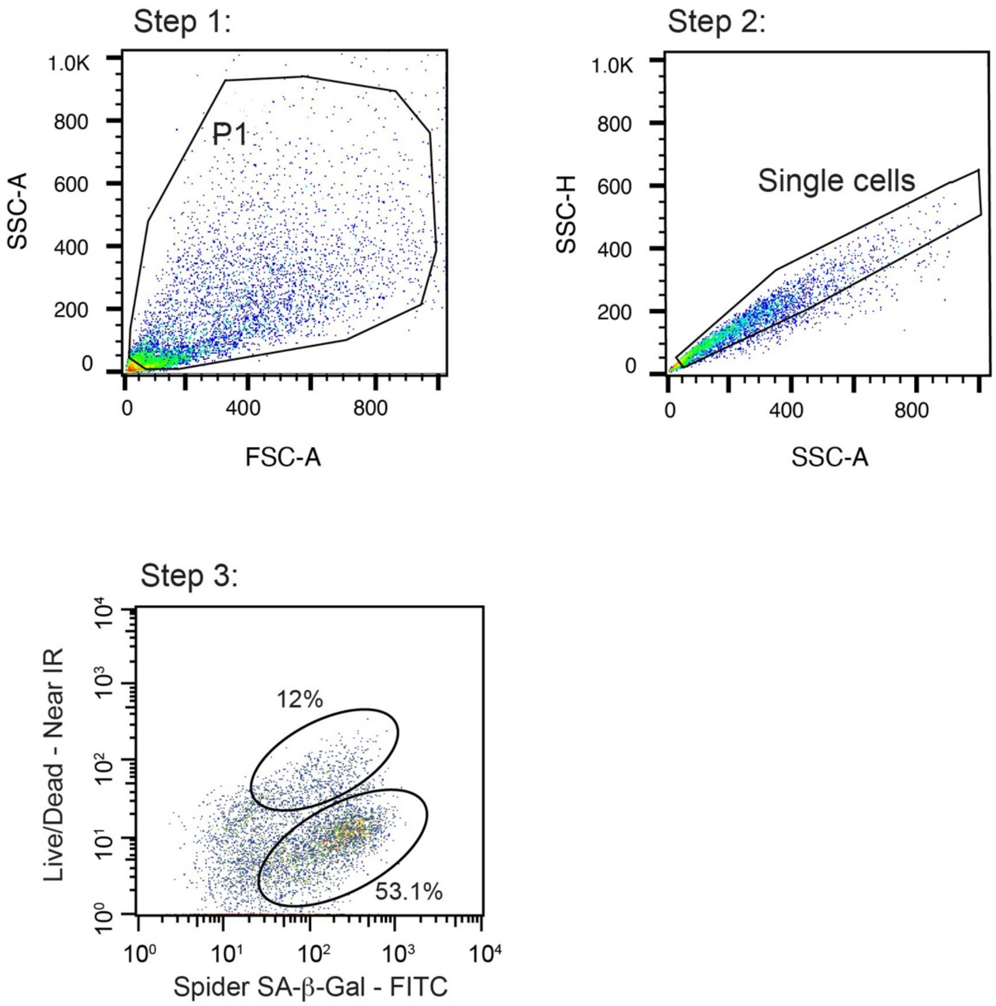

Supplementary Fig. 9 | Gating strategy used to identify live or dead senescent cells based on Spider SA- β-Gal staining.
