# Supplementary material for: METTL3-mediated chromatin contacts promote stress granule phase separation through metabolic reprogramming during senescence
Source: Nat Commun. 2024 Jun 26;15:5410. doi: 10.1038/s41467-024-49745-5 (PMC11208586; doi:10.1038/s41467-024-49745-5)
Supplement: Supplementary file 3 — Description of Additional Supplementary Files [file 41467_2024_49745_MOESM3_ESM.pdf]

Description of Additional Supplementary Files

Supplementary Data 1 List of primers used in the current study.

Supplementary Data 2 Spectral data for metabolomics identified in isotope tracing experiments.
