# Supplementary material for: METTL3-mediated chromatin contacts promote stress granule phase separation through metabolic reprogramming during senescence
Source: Nat Commun. 2024 Jun 26;15:5410. doi: 10.1038/s41467-024-49745-5 (PMC11208586; doi:10.1038/s41467-024-49745-5)
Supplement: Supplementary file 6 — Reporting Summary [file 41467_2024_49745_MOESM6_ESM.pdf]

nature portfolio

Corresponding author(s): Rugang Zhang

Last updated by author(s): Apr 22, 2024

# Reporting Summary

Nature Portfolio wishes to improve the reproducibility of the work that we publish. This form provides structure for consistency and transparency in reporting. For further information on Nature Portfolio policies, see our Editorial Policies and the Editorial Policy Checklist.

## Statistics

For all statistical analyses, confirm that the following items are present in the figure legend, table legend, main text, or Methods section.

| n/a | Confirmed | |
|---|---|---|
| ☐ | ☒ | The exact sample size (*n*) for each experimental group/condition, given as a discrete number and unit of measurement |
| ☐ | ☒ | A statement on whether measurements were taken from distinct samples or whether the same sample was measured repeatedly |
| ☐ | ☒ | The statistical test(s) used AND whether they are one- or two-sided<br>*Only common tests should be described solely by name; describe more complex techniques in the Methods section.* |
| ☒ | ☐ | A description of all covariates tested |
| ☒ | ☐ | A description of any assumptions or corrections, such as tests of normality and adjustment for multiple comparisons |
| ☐ | ☒ | A full description of the statistical parameters including central tendency (e.g. means) or other basic estimates (e.g. regression coefficient) AND variation (e.g. standard deviation) or associated estimates of uncertainty (e.g. confidence intervals) |
| ☐ | ☒ | For null hypothesis testing, the test statistic (e.g. *F*, *t*, *r*) with confidence intervals, effect sizes, degrees of freedom and *P* value noted<br>*Give P values as exact values whenever suitable.* |
| ☒ | ☐ | For Bayesian analysis, information on the choice of priors and Markov chain Monte Carlo settings |
| ☒ | ☐ | For hierarchical and complex designs, identification of the appropriate level for tests and full reporting of outcomes |
| ☐ | ☒ | Estimates of effect sizes (e.g. Cohen's *d*, Pearson's *r*), indicating how they were calculated |

*Our web collection on statistics for biologists contains articles on many of the points above.*

## Software and code

Policy information about availability of computer code

| | |
|---|---|
| Data collection | The amplification signal of qPCR data was acquired by QuantStudio"' Software VI.3. |
| Data analysis | Prism 9.2.0 was used for calculating p values. HiC-Pro 3.1.0 was used to align the reads to the hgl9 human genome, hichipper 0.7.7 were used for loop calling. Bowtie2 2.2.9 was used for genome alignment. Deeptools 3.3.1 was used for the generation of bigwig files. HOMER 4.10 was used for RPKM calculation. R 4.2.0 was used to generate mean density profiles. DEseq2 1.12.3 was used for estimation of the significance of differential fene expression among groups. NIH ImageJ 1.48v was used for image analysis. All codes are available from the corresponding author on reasonable request. |

For manuscripts utilizing custom algorithms or software that are central to the research but not yet described in published literature, software must be made available to editors and reviewers. We strongly encourage code deposition in a community repository (e.g. GitHub). See the Nature Portfolio guidelines for submitting code & software for further information.

## Data

Policy information about availability of data

All manuscripts must include a data availability statement. This statement should provide the following information, where applicable:

- Accession codes, unique identifiers, or web links for publicly available datasets
- A description of any restrictions on data availability
- For clinical datasets or third party data, please ensure that the statement adheres to our policy

HiChIP-seq, fastGRO–seq, KAS-seq, ATAC-seq, and RNA-seq data and processed files generated in this study have been deposited in the GEO under the accession number GSE243047 (HiChIP-seq, fastGRO–seq and KAS-seq, [https://www.ncbi.nlm.nih.gov/geo/query/acc.cgi?acc=GSE243047]), GSE243630 (ATAC-seq, [https://www.ncbi.nlm.nih.gov/geo/query/acc.cgi?acc=GSE243630]), and GSE243906 (RNA-seq, [https://www.ncbi.nlm.nih.gov/geo/query/acc.cgi?acc=GSE243906]). In addition, publicly available dataset such as H3K27Ac ChIP-seq (GSE74328 [https://www.ncbi.nlm.nih.gov/geo/query/acc.cgi?acc=GSE74328]), METTL3 and METTL14 Cut&Run-seq (GSE141992 [https://www.ncbi.nlm.nih.gov/geo/query/acc.cgi?acc=GSE141992]), senescent m6A-seq (GSE141993 [https://www.ncbi.nlm.nih.gov/geo/query/acc.cgi?acc=GSE141993]), primate m6A-seq (CRA005942 [https://ngdc.cncb.ac.cn/gsa/browse/CRA005942]) , senescent RNA-seq (GSE141994 [https://www.ncbi.nlm.nih.gov/geo/query/acc.cgi?acc=GSE141994]), mouse skeletal muscle tissue scRNA-seq (GSE197017 [https://www.ncbi.nlm.nih.gov/geo/query/acc.cgi?acc=GSE197017]), and Hi-C (GSE118494 [https://www.ncbi.nlm.nih.gov/geo/query/acc.cgi?acc=GSE118494]) were obtained from GEO. Metabolomics raw data has been deposited to Metabolomics Workbench (study_id: ST003182 [http://dx.doi.org/10.21228/M8NQ82]). Spectral data for metabolomics can be found in Supplementary Data 2.Source data for unprocessed immunoblots for Fig.3d, 3f, 4a, and Supplementary Fig. 1d and source data used for statistical analyses have been provided as Source data files.

## Research involving human participants, their data, or biological material

Policy information about studies with human participants or human data. See also policy information about sex, gender (identity/presentation), and sexual orientation and race, ethnicity and racism.

| | |
|---|---|
| Reporting on sex and gender | N/A |
| Reporting on race, ethnicity, or other socially relevant groupings | N/A |
| Population characteristics | N/A |
| Recruitment | N/A |
| Ethics oversight | N/A |

Note that full information on the approval of the study protocol must also be provided in the manuscript.

# Field-specific reporting

Please select the one below that is the best fit for your research. If you are not sure, read the appropriate sections before making your selection.

☒ Life sciences ☐ Behavioural & social sciences ☐ Ecological, evolutionary & environmental sciences

For a reference copy of the document with all sections, see nature.com/documents/nr-reporting-summary-flat.pdf

# Life sciences study design

All studies must disclose on these points even when the disclosure is negative.

| | |
|---|---|
| Sample size | For in vivo experiments, the sample size was estimated based on results from in vitro experiments and our previous experience with consideration of both sufficient statistical power and ethical use of animals. No statistical test was used to pre-determine the sample sizes. |
| Data exclusions | There was no exclusion from the experiments. |
| Replication | Experiments were repeated 3 times independently unlesss otherwise stated in figure legends and similar results were obtained. |
| Randomization | Experiments were all randomized. |
| Blinding | The investigators were blinded to group allocation during data collection. |

# Reporting for specific materials, systems and methods

We require information from authors about some types of materials, experimental systems and methods used in many studies. Here, indicate whether each material, system or method listed is relevant to your study. If you are not sure if a list item applies to your research, read the appropriate section before selecting a response.

## Materials & experimental systems

| n/a | Involved in the study | |
|---|---|---|
| ☐ | ☒ | Antibodies |
| ☐ | ☒ | Eukaryotic cell lines |
| ☒ | ☐ | Palaeontology and archaeology |
| ☐ | ☒ | Animals and other organisms |
| ☒ | ☐ | Clinical data |
| ☒ | ☐ | Dual use research of concern |
| ☒ | ☐ | Plants |

## Methods

| n/a | Involved in the study | |
|---|---|---|
| ☐ | ☒ | ChIP-seq |
| ☐ | ☒ | Flow cytometry |
| ☒ | ☐ | MRI-based neuroimaging |

# Antibodies

| | |
|---|---|
| Antibodies used | For HiChIP: anti-H3K27Ac (Abeam #4729); anti-METTL3 (Abeam #195352). For immunofluorescence, anti-YTHDFl (Proteintech, #17479-1-AP; 1:150); anti-YTHDF2 (Abeam, #245129 ; 1:150); anti-YTHDF3 (Proteintech, #25537-l-AP; 1:150); anti-TIAR (BD biosciences, #610352; 1:150); Alexa Fluor 488 conjugated Goat anti-Rabbit IgG (H+L) Cross-Adsorbed Secondary Antibody (Thermo, A-11008; 1:200);, Alexa Fluor 568 conjugated Goat anti-Mouse IgG (H+L) Cross-Adsorbed Secondary Antibody (Thermo, A11004; 1:200). For western blots: anti-RAS (Becton Dickinson, # 610001; 1:1,000); anti-p21(Abeam, # 7960; 1:1,000); anti-β-actin (CST#4967; 1:5,000); anti-METTL3 (Abeam # 195352; 1:1,000); anti-METTL14 (Sigma, #HPA038002; 1:1,000); anti-HK2 (abeam, #209847; 1:1,000), and anti-Flag (Sigma, #F1804; 1:2,000). |
| Validation | All antibodies were purchased from commercial vendors with validation information be available on the manufacturers' website. In addition, the validity of using anti-H3K27Ac and anti-METTL3 antibodies for ChIP-seq has been confirmed in studies referenced in Cancer Discov. 2016 Jun;6(6):612-29 and Nature Cell Biology volume 23, pages 355–365 (2021). Likewise, the suitability of anti-YTHDF1, anti-YTHDF2, anti-YTHDF3, and anti-TIAR antibodies for immunofluorescence has been verified in Nature Chemical Biology volume 16, pages 955–963 (2020). Additionally, the effectiveness of anti-RAS, anti-p21, anti-β-actin, anti-METTL3, anti-METTL14, anti-HK2, and anti-Flag antibodies for Western blotting has been established in Nature Cell Biology volume 23, pages 355–365 (2021) or accompanied with knockdown data. |

# Eukaryotic cell lines

Policy information about cell lines and Sex and Gender in Research

| | |
|---|---|
| Cell line source(s) | IMR90 cells were obtained from ATCC. 293T packaging cells were purchased from invitrogen. Phoenix packaging cells were obtained from Dr. Gary Nolan (Stanford University).Mouse NIH3T3 fibroblasts were obtained from Dr. Bin Tian (The Wistar Institute). |
| Authentication | Cell lines were re-authenticated by The Wistar Institute's Genomics Facility using short tandem repeat profiling using AmpFLSTR Identifiler PCR Amplification kit (Life Technologies). |
| Mycoplasma contamination | Regular Mycoplasma testing was performed using Lookout Mycoplasma PCR detection (Sigma). All cell lines used were tested negative for mycoplasma contamination. |
| Commonly misidentified lines (See ICLAC register) | No cell lines used in this study were found in the database of commonly misidentified cell lines that is maintained by ICLAC and NCBI Biosample. |

# Animals and other research organisms

Policy information about studies involving animals; ARRIVE guidelines recommended for reporting animal research, and Sex and Gender in Research

| | |
|---|---|
| Laboratory animals | Male young C57BL/6N (5-month old) and male aged C57BL/6J (21-month old) mice were obtained from Charles River facility.The mice were housed in a controlled environment with a 12-hour light/dark cycle, while the ambient temperature was maintained at 22-23°C with 40-60% humidity. |
| Wild animals | No wild animals were used in the study. |
| Reporting on sex | Male mice were obtained from Charles River facility. |
| Field-collected samples | No field-collected samples were used. |
| Ethics oversight | All procedures were performed by protocols and guidelines approved by the Institutional Animal Care and Use Committee (IACUC) at the Sanford Burnham Prebys (SBP) Medical Discovery Institute. |

Note that full information on the approval of the study protocol must also be provided in the manuscript.

## Plants

Seed stocks

*Report on the source of all seed stocks or other plant material used. If applicable, state the seed stock centre and catalogue number. If plant specimens were collected from the field, describe the collection location, date and sampling procedures.*

Novel plant genotypes

*Describe the methods by which all novel plant genotypes were produced. This includes those generated by transgenic approaches, gene editing, chemical/radiation-based mutagenesis and hybridization. For transgenic lines, describe the transformation method, the number of independent lines analyzed and the generation upon which experiments were performed. For gene-edited lines, describe the editor used, the endogenous sequence targeted for editing, the targeting guide RNA sequence (if applicable) and how the editor was applied.*

Authentication

*Describe any authentication procedures for each seed stock used or novel genotype generated. Describe any experiments used to assess the effect of a mutation and, where applicable, how potential secondary effects (e.g. second site T-DNA insertions, mosiacism, off-target gene editing) were examined.*

## ChIP-seq

### Data deposition

☒ Confirm that both raw and final processed data have been deposited in a public database such as GEO.

☒ Confirm that you have deposited or provided access to graph files (e.g. BED files) for the called peaks.

Data access links
*May remain private before publication.*

https://www.ncbi.nlm.nih.gov/geo/query/acc.cgi?acc=GSE243047.

Files in database submission

GSM7777865_H3K27Ac_HiChIP_proliferating.interactions.all.mango.txt.gz
GSM7777866_H3K27Ac_HiChIP_senescent_shcontrol.interactions.all.mango.txt.gz
GSM7777867_H3K27Ac_HiChIP_senescent_shMETTL3.interactions.all.mango.txt.gz
GSM7777868_H3K27Ac_HiChIP_senescent_shMETTL14.interactions.all.mango.txt.gz
GSM7777869_METTL3_HiChIP_proliferating.interactions.all.mango.txt.gz
GSM7777870_METTL3_HiChIP_senescent.interactions.all.mango.txt.gz

Genome browser session
(e.g. UCSC)

NA

### Methodology

Replicates

Experiments were performed with single replicate.

Sequencing depth

Aligned reads for H3K27Ac HiChIP were 29520171, 32575828, 40904862, 61554611for Control, Sen/shControl, Sen/shMETTL3 and Sen/shMETTL14 respectively. Valid intra-chromosomal paired-end tags (PETs) from METTL3 HiChIP were 48244038, 45505539 for Control and Senescence, respectively.

Antibodies

For HiChIP: anti-H3K27Ac (Abeam #4729); anti-METTL3 (Abeam #195352).

Peak calling parameters

hichipper 0.7.7 were used for loop calling with default parameters and with --peak-pad 700.

Data quality

Loops that passed FDR < 0.01with at least 4 reads and FDR < 0.05 were considered significant for H3K27Ac HiChIP and METTL3 HiChIP, respectively.

Software

HiC-Pro 3.1.0 was used to align the reads to the hg19 human genome. hichipper 0.7.7 were used for loop calling.

## Flow Cytometry

### Plots

Confirm that:

☒ The axis labels state the marker and fluorochrome used (e.g. CD4-FITC).

☒ The axis scales are clearly visible. Include numbers along axes only for bottom left plot of group (a 'group' is an analysis of identical markers).

☒ All plots are contour plots with outliers or pseudocolor plots.

☒ A numerical value for number of cells or percentage (with statistics) is provided.

### Methodology

Sample preparation

Samples were prepared as instructed by the manufacturers' manual. Briefly, 5X10A5 cells of each sample were washed with Hanks' HEPES buffer twice and incubate at 37°C for 15 min with1 mol/l SPiDER -gal working solution. Following the incubation, LIVE/DEAD™ Fixable Near-IR Dead Cell Stain Kit (ThermoFisher L34975) was used to stain live or dead cells at RT

| | |
|---|---|
| | for 15 min with protection from light. |
| Instrument | BD Biosciences, LSRII |
| Software | FlowJo version 10.0 |
| Cell population abundance | Senescent cells accounts for ~65% events for each senescent group. |
| Gating strategy | Gating was initially performed using FSC/SSC to identify live cells, followed by the removal of duplicate cells (SSC-A/SSC-H), and subsequent channel detection for FITC and Near-IR. |

☒ Tick this box to confirm that a figure exemplifying the gating strategy is provided in the Supplementary Information.
